# Astrocytic metabolic switch is a novel etiology for Cocaine and HIV-1 Tat-mediated neurotoxicity

**DOI:** 10.1038/s41419-018-0422-3

**Published:** 2018-03-16

**Authors:** Kalimuthusamy Natarajaseenivasan, Bianca Cotto, Santhanam Shanmughapriya, Alyssa A. Lombardi, Prasun K. Datta, Muniswamy Madesh, John W. Elrod, Kamel Khalili, Dianne Langford

**Affiliations:** 1 Department of Neuroscience, Lewis Katz School of Medicine at Temple University, Philadelphia, PA USA; 2 Department of Medical Genetics and Molecular Biochemistry and the Center for Translational Medicine, Lewis Katz School of Medicine at Temple University, Philadelphia, PA USA; 3 Center for Translational Medicine, Department of Pharmacology, Lewis Katz School of Medicine at Temple University, 3500 N Broad Street, Philadelphia, PA USA

## Abstract

Calcium (Ca^2+^) dynamics and oxidative signaling control mitochondrial bioenergetics in the central nervous system, where astrocytes are a major energy source for neurons. Cocaine use exacerbates HIV-associated neurocognitive disorders, but little is known about disruptions in astrocyte metabolism in this context. Our data show that the HIV protein Tat and cocaine induce a metabolic switch from glucose to fatty acid oxidation in astrocytes, thereby limiting lactate transport to neurons. Mechanistic analyses revealed increased Mitochondrial Ca^2+^ Uniporter (MCU)-mediated Ca^2+^ uptake in astrocytes exposed to Tat and cocaine due to oxidation of MCU. Since our data suggest that mitochondrial oxidation is dependent in part on MCU-mediated Ca^2+^ uptake, we targeted MCU to restore glycolysis in astrocytes to normalize extracellular lactate levels. Knocking down MCU in astrocytes prior to Tat and cocaine exposure prevented metabolic switching and protected neurons. These findings identify a novel molecular mechanism underlying neuropathogenesis in HIV and cocaine use.

## Introduction

Cocaine use significantly increases the risk for becoming infected with HIV. In fact, a significant number of cocaine users are infected with HIV, and cocaine use in the presence of HIV infection exacerbates HIV-associated neurocognitive disorders (HAND)^1^. HIV infection of the CNS occurs early after initial infection and although macrophages and microglia are responsible in large part for productive infection in the CNS, the virus also infects astrocytes albeit to a lesser extent^2–4^. Studies have shown that cocaine enhances viral replication in astrocytes and increases viral protein-mediated cellular damage^5,6^. One such viral protein is Tat, which is released from infected cells in the CNS and can activate astrocytes and damage surrounding neurons. In this context, multiple studies have addressed the interplay of HIV proteins and cocaine in the pathogenesis of HAND^1^.

Astrocytes provide energetic support for neurons by direct metabolic coupling interactions^7,8^. Neurons have a robust aerobic metabolism, whereas, astrocytes rely primarily on the ATP generated through glycolysis followed by the release of lactate to the extracellular milieu^9–12^. Lactate released from astrocytes is taken up by neurons and serves as a key metabolite for neuronal aerobic metabolism to meet the high energetic demands of neurons^9,12^. Under normal circumstances, this system is tightly regulated, but in diseases with an inflammatory component, such as HIV infection or during cocaine use, astrocytes respond by becoming activated and therefore increase their own metabolic demands. Cellular responses of astrocytes to Tat or cocaine are metabolically expensive and these energy-demanding events likely stimulate aberrant mitochondrial metabolism in astrocytes.

Mitochondrial Ca^2+^ ([Ca^2+^]_m_) uptake is crucial for bioenergetics through its role in activating Ca^2+^-sensitive dehydrogenases to promote ATP synthesis^13–16^. [Ca^2+^]_m_ uptake is driven by the electrochemical gradient across the inner mitochondrial membrane (Δψ_m_) and facilitated by the highly selective mitochondrial Ca^2+^ uniporter (MCU)^17–19^. MCU is a hetero-oligomeric complex and is regulated by several other proteins^20–25^. We expected that exposure of astrocytes to recombinant Tat (rTat) and cocaine would increase MCU-mediated Ca^2+^ uptake, thereby facilitating increased mitochondrial metabolism. In this context, we found that blocking [Ca^2+^]_m_ uptake by genetically targeting MCU could restore the neurotrophic phenotype of astrocytes by increasing extracellular lactate that would then be available to neurons. Our study investigates for the first time the previously unexplored pathway of increased [Ca^2+^]_m_ uptake contributing to a metabolic switch in astrocytes exposed to the HIV-1 protein Tat and to cocaine, and identifies a novel contributor to the pathogenesis of HAND in the context of cocaine use.

## Materials and methods

###  Human primary astrocytes and neurons

Fetal brain tissue (gestational age, 16–18 weeks) was obtained from elective abortion procedures performed in full compliance with National Institutes of Health and Temple University ethical guidelines. The tissue was washed with cold Hanks balanced salt solution (HBSS), and meninges and blood vessels were removed. Tissue in HBSS was digested with 0.25% trypsin (Sigma Chemical Co., St. Louis, Mo.) or papain (20 mg/ml) for 30 min at 37 °C for isolation of glial and neurons, respectively. The digestion was neutralized with fetal bovine serum (FBS), and the tissue was further dissociated to obtain single-cell suspensions. For glial cultures, the cells were plated in mixed glial growth media (DME:F12 media supplemented with insulin, FBS, L-glutamine, and gentamicin). The mixed culture was maintained under at 37 °C 5% CO_2_ for 5 days, and the media was replaced to remove any cell debris. To enrich for astrocytes, flasks were placed on an orbital shaker for 14–18 h at 200 rpm at 37 °C 5% CO_2_. Detached cells constituted the microglial component of the culture and were removed. Astrocytes that remained after shaking were fed with astrocyte media consisting of DME:F12 media supplemented with insulin, FBS, L-glutamine, and gentamicin. For neurons, a single-cell suspension was plated at a density of ~1.8 × 10^6^ cells/ 60 mm dish coated with poly-D lysine in neurobasal media with B27 supplement, horse serum, and gentamicin (NM5). After ~2 h, media was removed and neurons were re-fed with neurobasal media. Twenty-four hours later, cultures were re-fed with a complete change of neurobasal media without horse serum (NM0). Four days later, one fourth of the media was removed and replaced with NM0 supplemented with FDU and uridine. Purity of specific cell types was assessed by immunolabeling with anti-GFAP (astrocytes) or anti-MAP2/neurofilament (neurons).

### Cellular co-cultures and treatments

Astrocytes were exposed to 50 ng/ml recombinant Tat (rTat, ImmunoDX LLC, Woburn, WA) and/or 5 μM cocaine hydrochloride (Sigma-Aldrich) for 48 h. Concentrations used were based on our previous publication^26^. Astrocytes not exposed to rTat or cocaine served as controls. In some experiments, astrocytes were cultured onto 0.4 µm pore trans-well inserts (COSTAR Corning, Kennebunk, ME) and neurons were cultured on the plate below, where cells could communicate via soluble factors, but direct contact was prevented. Using the trans-well system, astrocytes cultured onto the trans-well filter were exposed to rTat/cocaine.

### RNA interference

Astrocytes (0.5 × 10^6^/well) grown on six-well plates were transfected with pools of 5 distinct siRNAs against MCU (ON-TARGETplus SMARTpool, Dharmacon, USA) (50 nM) using RNAiMAX transfection reagent (Thermo Scientific). As a control, non-targeting/scrambled siRNA (Scr siRNA) duplexes (Dharmacon) were used. Twenty-four hours post-transfection, the cells were exposed to rTat/cocaine for 48 h.

### Metabolite analyses of conditioned media and cells

Lactate (MAK064, Sigma-Aldrich), LDH (MAK066, Sigma-Aldrich), pyruvate (MAK071, Sigma-Aldrich), and Acetyl-CoA (MAK039, Sigma-Aldrich) concentrations and PDH activity (Abcam) in media and in cells were measured using commercial kits following the manufacturer’s instructions.

### ATP Measurement

Total ATP was assessed using CellTiter-Glo luminescent assay (Promega) according to the manufacturer’s instructions^20,22,27,28^.

### Mitochondrial oxygen consumption rate and superoxide measurements

Intact astrocytes treated with rTat/cocaine for 48 h were subjected to oxygen consumption rate (OCR) measurement at 37 °C in an XF96 extracellular flux analyzer (Seahorse Bioscience). Astrocytes (0.5 × 10^5^) were sequentially challenged with 2 µM oligomycin, 0.5 µM FCCP, and 0.5 µM rotenone plus antimycin A to measure basal and maximal respiration, ATP production, proton leak, spare respiratory capacity, and non-mitochondrial respiration^29^. For measuring the extracellular acidification rate (ECAR), rTat/cocaine-treated astrocytes were sequentially challenged with 10 mM glucose, 2 µM oligomycin, and 100 mM 2DG to measure glycolysis, glycolytic capacity and to calculate glycolytic and non-glycolytic acidification. Measurement of fatty acid oxidation (β-Oxidation OCR) in treated astrocytes was performed as previously described^29^. Mitochondrial superoxide was measured using the mitochondrial oxygen free radical indicator MitoSOX Red (Molecular Probes; Invitrogen) as described previously^30,31^.

### Simultaneous measurement of Ca^2+^ Uptake and Δψ_m_ in permeabilized cell system

Astrocytes treated with rTat/cocaine were washed in Ca^2+^ free PBS, pH 7.4. An equal amount of cells (7 × 10^6^ cells) from all treatment conditions were suspended and permeabilized with 40 μg/ml digitonin in 1.5 ml of intracellular medium (ICM) composed of 120 mM KCl, 10 mM NaCl, 1 mM KH_2_PO_4_, 20 mM HEPES-Tris, pH 7.2, and 2 μM thapsigargin to block the sarcoplasmic/endoplasmic reticulum calcium ATPase (SERCA) pump. All measurements were performed in the presence of 5 mM succinate. The simultaneous measurement of Δψ_m_ and extra-mitochondrial Ca^2+^ ([Ca^2+^]_out_) clearance as an indicator of [Ca^2+^]_m_ uptake was achieved by loading the permeabilized cells with JC-1 (800 nM) and Fura2-FF (0.5 μM), respectively. Mitochondrial uncoupler, Carbonyl cyanide m-chlorophenyl hydrazone (CCCP) was added as indicated to collapse the mitochondrial membrane potential. Fluorescence was monitored in a multi-wavelength excitation dual-wavelength emission fluorimeter (Delta RAM, PTI) as described previously^20,22,27,28^.

### Cytosolic Ca^2+^ dynamics

Astrocytes grown on 25 mm Cell-Tak (BD Biosciences, Bedford, MA) coated glass coverslips were treated with rTat/cocaine for 48 h. After 48 h, the astrocytes were loaded with Fluo-4/AM for 30 min for measurements of [Ca^2+^]_c_ as described previously . After 1 min of baseline recording, glutamate (200 μM) was added, and confocal images were recorded every 3 s (510 Meta; Carl Zeiss, Inc.) at 561 nm excitation using a 63 × oil objective. Images were quantitated by ImageJ (NIH)^32–34^.

### Mitochondrial Ca^2+^ dynamics

Astrocytes grown on 25 mm Cell-Tak (BD Biosciences, Bedford, MA) coated glass coverslips were treated with rTat/cocaine for 48 h. After 48 h, astrocytes were loaded with rhod-2/AM for 50 min for measurements of [Ca^2+^]_m_ as described previously . After 1 min of baseline recording, glutamate (200 μM) or ionomycin (2.5 μM) was added, and confocal images were recorded every 3 s (510 Meta; Carl Zeiss, Inc.) at 561 nm excitation using a 63 × oil objective. Images were quantitated by ImageJ (NIH)^32–34^.

### MCU-mPEG Gel-Shift Assay

Astrocytes expressing MCU-FLAG (AdMCU) were treated with rTat/cocaine for 48 h and assessed by the mPEG assay as described previously^35^. Astrocytes were infected with AdMCU and 48h post-infection, total cell proteins were precipitated with 10% w/v trichloroacetic acid (TCA) in acetone. TCA precipitated samples were dissolved in strong denaturing buffer (DB) (200 mM Tris-HCl (pH 8.5), 10 mM EDTA, 0.5% SDS and 6 M Urea). The samples were then subjected to 0.4 mM methoxypolyethylene glycol (MW 5 kDa; mPEG5)-maleimide incubation for 30 min. mPEG5-conjugated MCU was resolved on SDS-PAGE and probed with anti-FLAG antibody to visualize the molecular weight shift.

### Western blotting

Cell extracts were prepared from rTat/cocaine-treated astrocytes and neurons using RIPA buffer (50 mM Tris-HCl, pH 7.4, 150 mM NaCl, 0.25% deoxycholic acid, 1 mM EDTA, 1% NP-40, protease inhibitor cocktail (Complete, Roche), and Halt phosphatase inhibitor cocktail (Thermo Scientific). Equal amounts of protein (25 μg/lane) were separated on 4–12% Bis-Tris polyacrylamide gel, transferred to a nitrocellulose membrane using iBlot 2 NC regular stacks (Thermo Scientific), and probed with antibodies specific for MCT4 (1:1000, Santa Cruz), PDH E1 alpha subunit (phospho S293) (1:500, Abcam), PDH (1:1000, Abcam), OXPHOS (1:1000, Abcam), FLAG (1:2000, Sigma Aldrich), p-CAMKII (1:1000, Abcam), CAMKII (1:1000, Abcam), p-AMPK (1:1000, Cell Signaling Technology), AMPK (1:1000, Cell Signaling Technology), p-ACC (1:1000, Cell Signaling Technology), ACC (1:1000, Cell Signaling Technology), FAS (1:1000, Cell Signaling Technology), CPT1 (1:1000, Abcam), CPT2 (1:1000, Abcam), MCU (1:500 Sigma Aldrich), Synaptophysin (1:1000, Abcam), MAP2 (1:1000, Cell Signaling), PSD95 (1:1000, Abcam), β-actin (1:4000, Santa Cruz), Oxa1 (1:1000, Santa Cruz), GAPDH (1:1000, Santa Cruz), and TOM20 (1: 5000, Santa Cruz).

### Immunofluorescence Labeling

Following treatment with rTat/cocaine, astrocytes alone or astrocytes co-cultured with neurons plated onto glass coverslips were fixed in PBS containing 4% (w/v) paraformaldehyde for 20 min at room temperature. The cells were permeabilized with PBS containing 3% bovine serum albumin and 0.5% (v/v) Triton X-100 for 15 min at room temperature and then incubated with anti-GFAP antibody (1:200 dilution; Abcam)/anti-CPT1A (1:200; Abcam) or anti-GFAP/anti-MAP2 (1:200, Abcam) overnight at 4 °C. After 3 washes with PBS, the cells were incubated for 1 h with Alexa Fluor 568-conjugated anti-rabbit IgG (Abcam; 1:200 dilution) or Alexa Fluor 488-conjugated anti-mouse antibody (1:200 dilution), washed with PBS, and mounted with Vectashield containing DAPI (Vector Lab., Burlingame, CA, USA). The samples were visualized by fluorescence microscopy (Leica, Buffalo Grove, IL, USA) and fluorescence intensity of CPT1A/MAP2 was quantified using image J software.

### Enzyme-Linked Immunosorbent Assay (ELISA)

Cell culture supernatants from rTat, cocaine, or rTat and cocaine-treated astrocytes co-cultured with primary neurons were analyzed by ELISA for levels of secreted TNF-α and IL-6. Any non-adherent cells were removed by centrifugation and the remaining supernatants were analyzed using a human TNF-α ELISA kit (Invitrogen) and human IL-6 ELISA kit (Thermo Scientific) according to the manufacturers’ instructions.

### Statistical analyses

Data from multiple experiments (≥3) were quantified and expressed as Mean ± SE, and differences between groups were analyzed by using two-tailed paired Student’s *t*-test or, when not normally distributed, a nonparametric Mann–Whitney *U-*test was used. Differences in means among multiple datasets were analyzed using one-way ANOVA with the Kruskal–Wallis test, followed by pairwise comparison using the Dunn test. A *P* ≤ 0.05 was considered significant in all analyses. The data were computed either with GraphPad Prism version 7.0 or SigmaPlot 11.0 software.

## Results

**Fig. 1:**
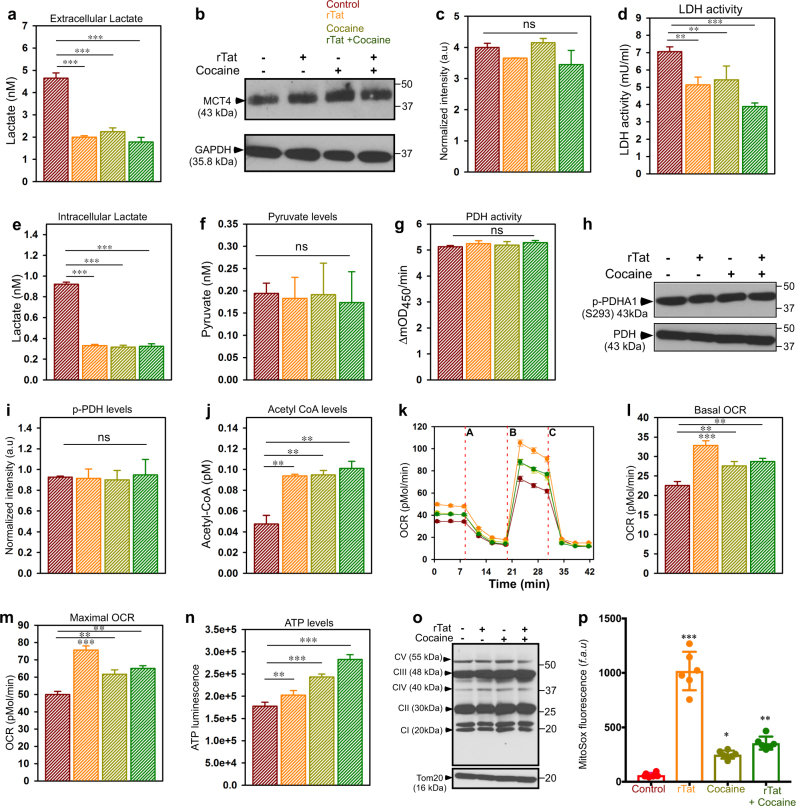
Exposure of astrocytes to rTat/cocaine augment mitochondrial respiration. **a** Extracellular lactate levels in media from astrocytes exposed to rTat, cocaine, or rTat and cocaine. **b** Representative Western blot of monocarboxylate transporter 4 (MCT4) levels in astrocytes exposed to rTat, cocaine, or rTat and cocaine. **c** Quantification of normalized intensity of MCT4 levels from (**b**) to GAPDH. **d** Quantification of LDH activity in astrocytes exposed to rTat, cocaine, or rTat and cocaine. **e** Quantification of intracellular lactate levels in astrocytes exposed to rTat, cocaine, or rTat and cocaine. **f** Quantification of pyruvate levels in astrocytes exposed to rTat, cocaine, or rTat and cocaine. **g** Quantification of PDH activity in astrocytes exposed to rTat, cocaine, or rTat and cocaine. **h** Representative western blot of PDHA1 (phospho S293) and PDH from astrocytes exposed to rTat, cocaine, or rTat and cocaine. **i** Quantification of normalized PDHA1 (phospho S293) levels to total PDH. **j** Quantification of Acetyl-CoA levels in astrocytes exposed to rTat, cocaine, or rTat and cocaine. **k** Measurement of oxygen consumption rate (OCR) in astrocytes exposed to rTat, cocaine, or rTat and cocaine. After basal OCR measurement, oligomycin (A), FCCP (B), and rotenone + Antimycin A (C) were added as indicated. Representative traces of OCR in astrocytes are shown. **l**, **m** Quantification of basal OCR (**l**), maximal OCR (**m**) in astrocytes exposed to rTat, cocaine, or rTat and cocaine. **n** Quantification of cellular ATP levels in astrocytes exposed to rTat, cocaine, or rTat and cocaine. **o** Representative Western blot for electron transport chain complex components (CI, CII, CIII, CVI, CV) in astrocytes exposed to rTat, cocaine, or rTat and cocaine. The outer mitochondrial membrane receptor, TOM20 was used as the loading control. **p** Quantification of mitochondrial ROS levels in astrocytes exposed to rTat, cocaine, or rTat and cocaine. Data indicate Mean ± SEM; ***P < 0.001, ** *P* < 0.01, *P < 0.05; *n* = 12–16

To evaluate whether exposure of astrocytes to rTat/cocaine affects lactate metabolism, extracellular lactate levels were measured in media from astrocytes exposed to rTat and/or cocaine. Exposure of astrocytes to rTat, cocaine, and rTat/cocaine significantly decreased extracellular lactate levels (Fig. 1a). Since lactate is transported between the intra and extracellular compartments by the monocarboxylate transporters (MCT), we next asked whether decreased extracellular lactate levels were a result of decreased astrocytic MCT4 expression. Western blot analysis showed no change in MCT4 in astrocytes exposed to rTat, cocaine, or rTat/cocaine (Fig. 1b, c), suggesting no defect in the efflux of lactate from astrocytes. Because we observed a decrease in extracellular lactate levels with no change in MCT4 expression, we anticipated decreased lactate production and lactate dehydrogenase (LDH) activity in astrocytes exposed to rTat/cocaine. Exposure of astrocytes to rTat, cocaine, and rTat/cocaine decreased LDH activity with the greatest decrease observed in the combined treatment (Fig. 1d). Consistent with decreased LDH activity, we observed decreased intracellular lactate levels as well (Fig. 1e). Next, we asked whether the decrease in LDH activity and lactate were due to impaired glucose metabolism leading to less available pyruvate for LDH enzyme activation. Surprisingly, the ECAR and glycolysis remained unchanged following rTat/cocaine exposure (Supplementary Figure 1a-e). In line with the stable glycolytic functioning of the astrocytes exposed to rTat/cocaine, pyruvate levels remained unaltered as well (Fig. 1f).

We then asked whether stable glucose metabolism and unchanged pyruvate levels with decreased lactate production in astrocytes could be a result of a metabolic switch in astrocytes from anaerobic to aerobic glycolysis and mitochondrial metabolism. The pyruvate dehydrogenase (PDH) complex links glycolysis with oxidative phosphorylation (OxPhos) converting pyruvate to Acetyl-CoA for entry into the tricarboxylic acid cycle. Calcium-mediated activation of PDH phosphatase dephosphorylates the PDH e1α subunit to activate the PDH complex. Thus, we assessed PDH activity and phosphorylation levels in astrocytes exposed to rTat/cocaine. The PDH activity remained unchanged between control and treated astrocytes (Fig. 1g) with no changes in levels of p-PDH (Fig. 1h, i). However, we observed elevated levels of Acetyl-CoA in astrocytes exposed to rTat, cocaine, and rTat/cocaine (Fig. 1j). In addition, astrocytes exposed to rTat/cocaine showed increased OCR (Fig. 1k). The basal respiration in astrocytes exposed to rTat/cocaine was increased (Fig. 1l) as well, suggesting increased mitochondrial metabolism. An increase was observed in the maximal respiratory capacity (Fig. 1m), which resulted in much higher spare capacity and ATP coupled respiration (Supplementary Figs. 1f and 1g). In line with increased mitochondrial respiration, astrocytes exposed to rTat/cocaine had elevated ATP levels (Fig. 1n). However, neither the increased OCR nor the ATP generation were due to changes in mitochondrial respiratory complex components (Fig. 1o). Furthermore, increased mitochondrial respiration was also marked by increased mitochondrial reactive oxygen species (mROS) generation as quantified by MitoSOX red fluorescence (Fig. 1p). Taken together, activation of astrocytes with rTat/cocaine induces mitochondrial respiration.

**Fig. 2:**
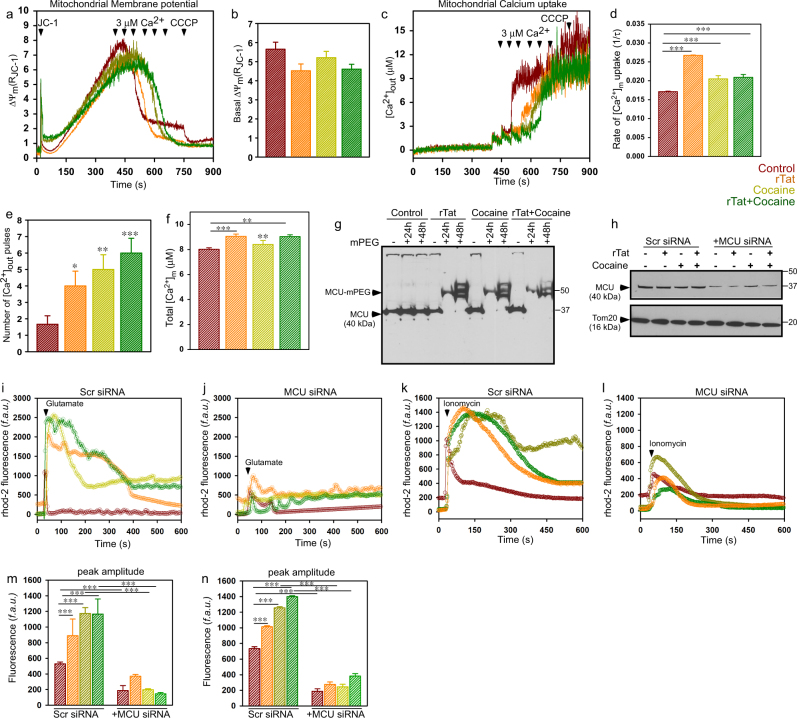
MCU-mediated Ca^2+^ uptake enhances mitochondrial metabolism in astrocytes exposed to rTat/cocaine. **a**,** c** Representative Δψ_m_ (**a**) and [Ca^2+^]_out_ traces (**c**). Permeabilized cells were loaded with the Δψ_m_ indicator JC-1 and extra-mitochondrial Ca^2+^ ([Ca^2+^]_out_) indicator Fura2FF to which a series of extra-mitochondrial Ca^2+^ pulses (3 μM) were added (arrowheads) to assess the [Ca^2+^]_out_ clearance rate. **b** Quantification of basal Δψ_m_ before the addition of [Ca^2+^]_out_. **d–f** Quantification of the rate of [Ca^2+^]_m_ uptake as a function of decrease in [Ca^2+^]_out_ (**d**), number of extra-mitochondrial Ca^2+^ pulses handled (**e**), Total [Ca^2+^]_m_ taken up by astrocytes treated with rTat, cocaine, or rTat and cocaine (**f**). **g** rTat/cocaine-induced oxidative modification of MCU. Representative Western blot of lysates prepared from astrocytes exposed to rTat, cocaine, or rTat and cocaine and expressing MCU-FLAG (AdMCU). The lysates were incubated with mPEG5 (30 mins) and oxidation of MCU was identified using anti-Flag antibody. **h** Cell lysates prepared from control (Scr siRNA) and MCU KD astrocytes exposed to rTat, cocaine, or rTat and cocaine were assessed by Western blotting with anti-MCU antibody. The outer mitochondrial membrane receptor, TOM20 was used as the loading control. **i**,** j** Mean traces of [Ca^2+^]_m_ (rhod-2) dynamics in control (Scr siRNA) (**i**) and MCU KD (**j**) astrocytes exposed to rTat, cocaine, or rTat and cocaine. After measurement of baseline fluorescence, cells were stimulated with glutamate (200 μM, arrowheads) and changes in [Ca^2+^]_m_ fluorescence were measured. **k, l** Mean traces of [Ca^2+^]_m_ (rhod-2) dynamics in control (Scr siRNA) (**k**) and MCU KD (**l**) astrocytes exposed to rTat, cocaine, or rTat and cocaine. After measurement of baseline fluorescence, cells were stimulated with Ionomycin (2.5 μM, arrowheads) and changes in [Ca^2+^]_m_ fluorescence were measured. **m** Quantification of peak amplitude of rhod-2 fluorescence after glutamate stimulation. **n** Quantification of peak amplitude of rhod-2 fluorescence after ionomycin stimulation. Data represents Mean ± SEM; *P < 0.05, **P < 0.01, ***P < 0.001; *n* = 6-8. Scr siRNA is a Scrambled/non-target siRNA control.

Increased mROS generally correlates with opening the permeability transition pore (PTP) and cell death^16^. To assess whether rTat/cocaine treatment induces PTP opening, we simultaneously measured the mitochondrial Ca^2+^ retention capacity (CRC) and Δψ_m_ by loading astrocyte with JC1 (Δψ_m_ indicator) and Fura FF (Ca^2+^ indicator). Astrocytes exposed to rTat/cocaine were permeabilized and a series of 3 μM extra-mitochondrial Ca^2+^ pulses (arrowheads) were added to measure the rate of [Ca^2+^]_m_ uptake (Fig. 2a, c). Astrocytes exposed to rTat and/or cocaine showed increased CRC (Fig. 2c, e) with sustained membrane potential compared to control astrocytes (Fig. 2a). No change was observed in basal Δψ_m_ (Fig. 2b). Astrocytes treated with rTat/cocaine had increased [Ca^2+^]_m_ uptake rate (calculated from first Ca^2+^) (Fig. 2d) and mitochondrial matrix Ca^2+^ (calculated as sum of area under the curve of all the Ca^2+^ pulses handled) (Fig. 2f).

Next, we asked whether the increased [Ca^2+^]_m_ uptake rate and accumulated matrix Ca^2+^ was due to increased activity of MCU. [Ca^2+^]_m_ uptake is mediated by MCU and a recent study has shown that the pore-forming subunit, MCU can be oxidized by luminal ROS^35^. Thus, we asked whether increased mROS generated in astrocytes exposed to rTat/cocaine could oxidize MCU and increase [Ca^2+^]_m_ uptake. To assess if changes in [Ca^2+^]_m_ dynamics were due to MCU oxidation, we performed MCU-mPEGylation assays in astrocytes expressing MCU-Flag (AdMCU)^35^. Astrocytes exposed to rTat, cocaine, and rTat/cocaine elicited robust mPEGylation of MCU in a time dependent manner (Fig. 2g). Collectively, these results suggest that the mROS oxidizes MCU and promotes [Ca^2+^]_m_ uptake, which in turn increases mitochondrial metabolism during stress. Next, we assessed whether increased mitochondrial respiration in astrocytes exposed to rTat and cocaine is mediated by an increase in the rate of [Ca^2+^]_m_ uptake. It has been shown that cocaine/ionomycin treatment stimulates mitochondrial metabolism in a Ca^2+^-dependent manner^36^. Since we hypothesized MCU-mediated Ca^2+^ uptake as a nodal point for the astrocyte metabolic switching during rTat/cocaine stimulation, we assessed if blocking MCU-mediated [Ca^2+^]_m_ uptake in astrocytes could prevent the decreased lactate and increased Acetyl-CoA. For these experiments, we adopted an RNAi-mediated MCU knock down (KD) strategy (Fig. 2h). Twenty-four hours post-transfection, astrocytes were exposed to rTat/cocaine and [Ca^2+^]_m_ dynamics (rhod-2 AM) were measured. After baseline measurement of [Ca^2+^]_m_ fluorescence, astrocytes were stimulated with glutamate (200 μM, arrowhead) or ionomycin (2.5 μM, arrowhead) and changes in [Ca^2+^]_m_ dynamics were measured (Fig. 2i–l). In control (Scr siRNA) astrocytes rTat/cocaine treatment resulted in a sustained elevation of [Ca^2+^]_m_ uptake after stimulation with glutamate/ionomcyin (Fig. 2i, k, m, n). On the contrary, transient KD of MCU in astrocytes reduced [Ca^2+^]_m_ uptake even with rTat/cocaine exposure (Fig. 2j, l, m, n).

**Fig. 3:**
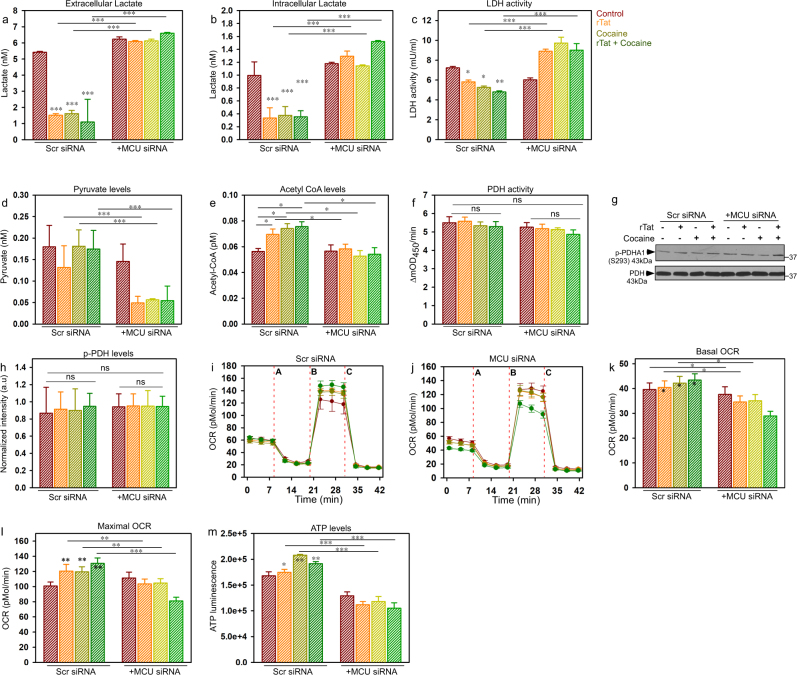
Knock down of MCU switches astrocytes treated with rTat/cocaine to utilization of fatty acids and facilitates glucose oxidation. **a** Quantification of extracellular lactate levels in control (Scr siRNA) and MCU KD astrocytes exposed to rTat, cocaine, or rTat and cocaine. **b** Quantification of intracellular lactate levels in control and MCU KD astrocytes exposed to rTat, cocaine, or rTat and cocaine. **c** Quantification of LDH activity in control and MCU KD astrocytes exposed to rTat, cocaine, or rTat and cocaine. **d** Quantification of pyruvate levels in control and MCU KD astrocytes exposed to rTat, cocaine, or rTat and cocaine. **e** Quantification of Acetyl-CoA levels in control and MCU KD astrocytes exposed to rTat, cocaine, or rTat and cocaine. **g** Cell lysates prepared from control and MCU KD astrocytes exposed to rTat, cocaine, or rTat and cocaine were assessed by Western blotting with antibodies specific for PDHA1 (phospho S293) and PDH. Quantification of normalized PDHA1 (phospho S293) levels with total PDH (h). **i** Measurement of OCR in control and MCU KD astrocytes exposed to rTat, cocaine, or rTat and cocaine. Representative traces of OCR in control (**i**) and MCU KD astrocytes (**j**). **k**, **l** Quantification of basal OCR (**k**), maximal OCR (**l**) in control and MCU KD astrocytes exposed to rTat, cocaine, or rTat and cocaine. **m** Quantification of cellular ATP levels in control and MCU KD astrocytes exposed to rTat, cocaine, or rTat and cocaine. Data indicate Mean ± SEM; ***P < 0.001, **P < 0.01, *P < 0.05; *n* = 24–30

In line with decreased [Ca^2+^]_m_ uptake, KD of MCU in astrocytes prevented rTat/cocaine-mediated decreases in intracellular and extracellular lactate compared to control (Scr siRNA) astrocytes (Fig. 3a, b). The increased lactate levels in KD MCU astrocytes were likely due to the increased LDH activity (Fig. 3c). The increase in LDH activity was marked by increased conversion of pyruvate to lactate and thus, decreased pyruvate accumulation in astrocytes where MCU was knocked down (Fig. 3d). Also, MCU KD astrocytes had decreased Acetyl-CoA levels (Fig. 3e), but no change in PDH activity (Fig. 3f) or p-PDH levels (Fig. 3g, h). In accordance with decreased Acetyl-CoA levels, MCU KD astrocytes showed a marked decrease in basal and maximal OCR (Fig. 3i–l), spare capacity, proton leak (Supplementary Figs. 2a and 2b), and ATP levels (Fig. 3m) with no changes in electron transport chain complex components (Supplementary Figs. 2c-2h). Collectively, MCU-mediated Ca^2+^ uptake determines mitochondrial respiration and astrocytic metabolism during rTat/cocaine-induced stress conditions and thus knocking down MCU reverts astrocytic metabolism from pyruvate back to lactate utilization.

**Fig. 4:**
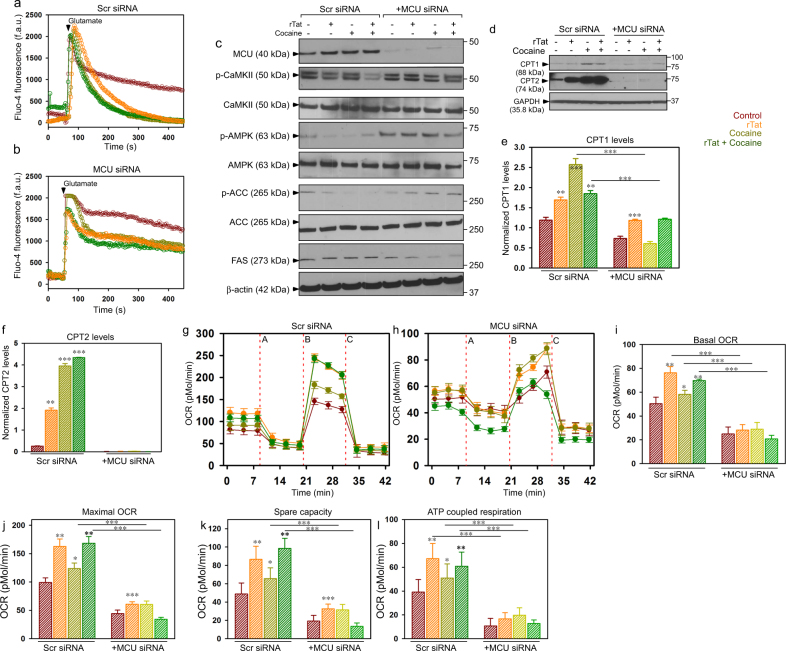
Mitochondrial fatty acid oxidation (FAO) is increased in astrocytes treated with rTat/cocaine. **a**, **b** Mean traces of [Ca^2+^]_c_ (Fluo-4) dynamics in control (Scr siRNA) (**a**) and MCU KD (**b**) astrocytes exposed to rTat, cocaine, or rTat and cocaine. After measurement of baseline fluorescence, cells were stimulated with glutamate (200 μM, arrowheads) and changes in [Ca^2+^]_c_ fluorescence were measured. **c** Cell lysates prepared from control and MCU KD astrocytes treated with rTat, cocaine, or rTat and cocaine were analyzed by Western blot with antibodies specific for p-CAMKII, CAMKII, p-AMPK, AMPK, p-ACC, ACC, FAS, and β-actin. **d** Cell lysates prepared from control and MCU KD astrocytes treated with rTat, cocaine, or rTat and cocaine were analyzed by Western blot with antibodies specific for CPT1, CPT2, and GAPDH. **e**, **f** Quantification of CPT1 (**e**) and CPT2 levels (**f**) normalized with GAPDH. **g** and **h** Measurement of OCR in control (**g**) and MCU KD (**h**) astrocytes with carnitine as a substrate. After basal OCR measurement, oligomycin (A), FCCP (B), and rotenone + antimycin A (C) were added as indicated. **i**–**l** Quantification of basal OCR (**i**), maximal OCR (**j**), spare capacity (**k**), and ATP-coupled respiration (**l**). Data indicate Mean ± SEM; ***P < 0.001, **P < 0.01, *P < 0.05; *n* = 12–16

It has been established that astrocytes express proteins involved in beta-oxidation and that they do not utilize fatty acids as their major energy reserve. Thus, we further hypothesized that during conditions of metabolic stress including rTat/cocaine stimulation, astrocytes may utilize fatty acids as an alternate energy source and that MCU KD would prevent the utilization of fatty acids and facilitates glucose oxidation in astrocytes. To illustrate MCU’s role in fatty acid oxidation (FAO), we speculated a relationship between MCU-mediated [Ca^2+^]_m_ uptake and AMPK-dependent modulation of lipid metabolism^37,38^. Because mitochondria play a key role in buffering cytosolic Ca^2+^ changes, we measured cytosolic Ca^2+^ transients (fluo4-AM) in astrocytes treated with rTat/cocaine. After baseline measurement of [Ca^2+^]_c_ fluorescence, astrocytes were stimulated with glutamate (200 μM, arrowhead) and [Ca^2+^]_c_ dynamics were measured (Fig. 4a). In line with increased MCU-mediated Ca^2+^ uptake in astrocytes treated with rTat/cocaine, stimulation with glutamate returned the [Ca^2+^]_c_ levels to baseline more rapidly than that of control astrocytes. (Fig. 4a). In contrast, knocking down MCU resulted in a phenotypic shift towards persistent elevation of [Ca^2+^]_c_ (Fig. 4b). The decrease or increase in [Ca^2+^]_c_ dynamics have opposing roles on CAMKII activation and AMPK phosphorylation (Fig. 4c). The increased [Ca^2+^]_c_ in MCU KD astrocytes resulted in CAMKII activation (Fig. 4c). Activation of CAMKII phosphorylated AMPK (p-AMPK), which in turn inactivated Acetyl-CoA carboxylase (ACC). The inhibition of ACC as a compensatory mechanism decreased fatty acid synthase (FAS) expression (Fig. 4c) and thus, could reduce the flux of substrates in the anabolic pathway and could negatively regulate FAO in MCU KD astrocytes.

Next, we examined carnitine palmitoyltransferase 1 (CPT1) and CPT2 levels as they are the rate limiting enzymes for beta-oxidation. We measured CPT1 and CPT2 levels in control (Scr siRNA) and MCU KD astrocytes exposed to rTat, cocaine, and rTat/cocaine. Parallel to the increased Acetyl-CoA levels in rTat/cocaine-treated astrocytes (Figs. 1j and 3e), the expression levels of both CPT1 and CPT2 were increased in astrocytes exposed to rTat, cocaine, and rTat/cocaine; whereas, ablation of [Ca^2+^]_m_ uptake by MCU KD significantly reduced CPT levels in astrocytes (Fig. 4d–f; Supplementary Fig. 3)

To investigate whether decreased CAMKII and AMPK activation and increased CPT levels were associated with increased FAO, carnitine was employed as a metabolic substrate and oxygen consumption rate was measured in control (Scr siRNA) and MCU KD astrocytes. Control (Scr siRNA) astrocytes treated with rTat/ cocaine had increased basal, maximal, mitochondrial reserve capacity, and ATP coupled respiration compared to MCU KD astrocytes (Fig. 4g–l). These data suggest that utilization of FAO by astrocytes exposed to rTat and cocaine generate increased ATP levels. Blocking MCU-mediated Ca^2+^ uptake prevented mitochondrial FAO in astrocytes exposed to rTat/cocaine.

**Fig. 5:**
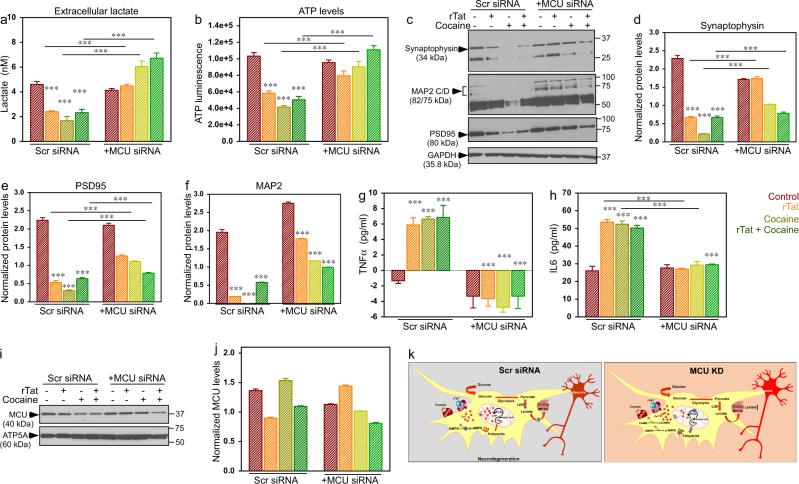
Co-culturing astrocytes knocked down for MCU protects neurons from rTat/cocaine-induced toxicity. **a** Quantification of extracellular lactate levels in control (Scr siRNA) and MCU KD astrocytes and neuronal co-cultured media. **b** Quantification of ATP levels in co-cultured neuronal lysate. **c** Lysates from neurons co-cultured with astrocytes were Western blotted for Synaptophysin, PSD-95 and MAP2. **d**–**f** Quantification of the normalized Synaptophysin (**d**), PSD-95 (**e**), and MAP2 (**f**) protein levels in neurons. **g**, **h** Quantification of cytokine levels in media from co-cultures: TNFα (**g**), and IL6 (**h**) levels. **i** Representative Western blot depicts unaltered MCU levels in neurons co-cultured with control (Scr siRNA) or MCU KD astrocytes. The mitochondrial membrane synthase, ATP5A was used as the loading control. **j** Quantification of normalized MCU levels in neurons co-cultured with control (Scr siRNA) or MCU KD astrocytes. Data represents Mean ± SEM; ***P < 0.001; *n* = 6–8. **k** Schematic representation of the proposed astrocytic metabolic switch results in neurodegeneration during rTat/cocaine treatment

Neuronal function is mediated in part by interactions with astrocytes that provide factors such as lactate to support neuronal fitness. In this context, we sought to identify whether the metabolic switch in astrocytes exposed to rTat and cocaine had direct effects on neuronal metabolism, synaptodendritic protein levels and cytokine production. To verify the effects of astrocytes exposed to rTat/cocaine on neurons, we adopted a trans-well co-culture strategy where astrocytes were plated in the upper chamber and neurons on the lower chamber. In this system, factors produced by astrocytes were able to contact neurons, but the two cell types were not in contact. Figure 5a, g, h reflect measures conducted in supernatant from the co-culture; whereas, Fig. 5b–f, i–j reflect levels of proteins in neuronal lysate. Similar to the effect of rTat/cocaine on astrocytes, we observed reduced extracellular lactate levels in media from astrocyte-neuron co-cultures exposed to rTat and cocaine (Fig. 5a). As previously shown, MCU KD in astrocytes prevented the decrease in extracellular lactate in response to rTat/cocaine exposure. Next, we asked whether decreases in the extracellular lactate levels impact neuronal energy production in the presence and absence of astrocyte MCU expression. Neuronal ATP levels were markedly decreased in neurons cultured with astrocytes that were exposed to rTat/cocaine (Fig. 5b), but returned to normal when MCU was knocked down in astrocytes. Next, we assessed the levels of pre- (synaptophysin) and post-(PSD-95) synaptic proteins as a measure of synaptic disruption in neurons. In line with decreased neuronal ATP levels, synaptophysin and PSD-95 protein levels were significantly reduced in neurons cultured with astrocytes exposed to rTat/cocaine compared to those exposed to rTat/cocaine with MCU KD (Fig. 5c–e). Since numerous studies have shown the involvement of MAP2 proteolytic dysfunction in neuronal cell death, we measured the MAP2 levels in neurons cultured with astrocytes exposed to rTat, cocaine, and rTat/cocaine with and without MCU KD. A significant reduction in MAP2 protein levels in neurons cultured with control (Scr siRNA) astrocytes exposed to rTat/cocaine was observed, suggesting decreased neuronal fitness and disruption of neuronal microtubules (Fig. 5c, f). Importantly, in neurons co-cultured with rTat/cocaine-treated astrocytes, where MCU was KD, MAP2 levels decreased less (Fig. 5c, f). To further investigate the cytoskeletal integrity, we performed immuno-fluorescent labeling of MAP-2 protein in neurons co-cultured with control and MCU KD astrocytes. Predominant MAP-2 labeling was observed in control neurons, whereas co-culturing with astrocytes exposed to rTat/cocaine decreased MAP2 immunofluorescence and increased neuronal cell death (Supplementary Figs. 4a and 4b). In neurons co-cultured with MCU KD astrocytes exposed to rTat/cocaine MAP-2 immunolabeling was preserved suggesting improved neuronal fitness (Supplementary Figs. 4c and 4d). Additionally, we evaluated the inflammatory factor levels in the media of astrocyte/neuron co-cultures where astrocytes were exposed to rTat/cocaine with and without MCU KD. Both TNFα and IL6 levels were significantly increased in media from astrocytes/neurons exposed to rTat/cocaine (Fig. 5g, h). Because we observed restoration of glycolysis in MCU KD astrocytes exposed to rTat/cocaine, we presumed that co-culturing MCU KD astrocytes and neurons exposed to rTat/cocaine could preserve neuronal function. The MCU levels remain unchanged in the neurons, so the observed metabolic changes in MCU KD astrocytes are due to modulation of MCU levels only in astrocytes (Fig. 5i, j). Collectively, neurons co-cultured with astrocytes with KD MCU-mediated Ca^2+^ uptake showed reduced toxicity to rTat/cocaine. Thus, the ability to alter astrocytic metabolism during inflammatory or neuronal stress conditions is crucial in preserving neuronal fitness (Fig. 5k).

## Discussion

Astrocytes use glucose as their main energy source and 60% of the glucose taken up is converted to lactate for delivery to neurons^39–42^. On the other hand, infection with HIV or inflammation as observed with cocaine abuse triggers astrocyte activation. Once astrocytes become reactive, their energy utilization is optimized to meet increased metabolic demands in response to challenge. Though several studies have proposed mechanisms for the involvement of astrocyte reactivity in HIV-cocaine-mediated neuropathogenesis, aberrant energy metabolism in astrocytes as a possible mechanism of neuropathogenesis is under studied.

The salient feature of the present study is that the hypometabolic state in the CNS during infection/inflammation will prompt astrocytes to utilize energy substrates for their own metabolism rather than transferring them to neurons. Reduction in extra and intracellular lactate levels (Fig. 1a, e) implicates a metabolic shift in astrocytes exposed to rTat/cocaine. Additionally, astrocytes have higher expression of PDH kinase machinery (PDK2 and PDK4) that phosphorylates and inactivates PDH complex, which explains unchanged PDH activity in astrocytes treated with rTat/cocaine^43^. Although we observed increased MCU-mediated Ca^2+^ uptake (Fig. 2), PDH activity and the phosphorylation state of PDH remain unaltered (Fig. 1g–i), establishing the main product of glucose oxidation to be pyruvate, and not Acetyl-CoA. Also, we expect a negligible role for PDH activity in astrocytic mitochondrial metabolism (Fig. 1g), which needs further study and validation. Because we observed increased Acetyl-CoA levels in rTat/cocaine-treated astrocytes (Fig. 1j), we anticipated an augmented formation of Acetyl-CoA in the mitochondrial matrix by the sequential oxidation of acyl-CoA or FAO (Fig. 4). Thus, enhancement of mitochondrial oxidation in astrocytes exposed to rTat/cocaine (Figs. 1, 3 and 4) provides evidence of an energy deficit for neurons by the loss of extracellular lactate release. Recent studies have shown cocaine to influence glucose metabolism consequently impacting various regions of the brain^44^. Several lines of evidence link increased fatty acid oxidation and inflammation in the CNS^45^. Accordingly, we showed FAO to play a significant role in sustaining inflammatory responses in the CNS and therapeutic interventions targeting FAO could well be appreciated. Cocaine exposure is expected to increase intracellular Ca^2+^ transients through G-protein coupled receptors (GPCR) stimulation^36^. Likewise, Tat is known to cause over activation of NMDA receptors and intracellular Ca^2+^-dependent damage to oligodendrocytes and myelin^46,47^. Because rTat/cocaine treatment increases intracellular Ca^2+^ dynamics by either GPCR or NMDA activation, we anticipated increased [Ca^2+^]_m_ uptake (Fig. 2) in astrocytes treated with rTat/cocaine.

Since [Ca^2+^]_m_ entry and mROS production are often interdependent events, the increased MCU-mediated Ca^2+^ uptake resulted in mROS generation (Fig. 1p).

Increased [Ca^2+^]_m_ and mROS generation are often associated with sensitization of the mitochondrial permeability transition pore (PTP) opening^35,62,63,]^. But surprisingly, we observed delayed opening of PTP in rTat/cocaine-treated astrocytes (Fig. 2a, c). This could well be explained by the modulation of the PTP by the substrates availability for mitochondrial energy^48^. It is well known that lower mitochondrial Ca^2+^ loads are required when electrons are provided to complex I rather than to complex II or IV^48^. These variations in the sensitivity of the PTP is independent of differences in membrane potential, matrix pH, Ca^2+^ uptake, oxidation–reduction status of pyridine nucleotides, or production of H_2_O_2_, but rather is directly related to the rate of electron flow through complex I. Oxidation of fatty acids by the mitochondrial β-oxidation system is the most important source of FADH_2_ generation. Despite being a potent source of FADH_2_, FAO by mitochondria will not exhibit the reverse electron transport (RET)-associated O_2_^−^ generation^51–59^, and thus delayed PTP opening in astrocytes treated with rTat/cocaine.

Increased mitochondrial luminal ROS positively oxidized MCU (Fig. 2i) and enhanced its activity. Oxidation of MCU is known to facilitate increased [Ca^2+^]_m_ uptake by undergoing glutathionylation at cysteine 96 of the MCU N-terminal domain (NTD). Furthermore, solution NMR spectroscopy showed that glutathione conjugated MCU underwent a conformational change within the NTD and appeared to promote persistent channel activity irrespective of its interaction with the gatekeeper, mitochondrial calcium uptake 1 (MICU1)^35^. On the other hand, since mitochondria are the site of FAO and [Ca^2+^]_m_ is the key player for mitochondrial bioenergetics, we speculated that blocking [Ca^2+^]_m_ would have an impact on FAO. Indeed, we observed that knocking down MCU resulted in decreased FAO in astrocytes (Fig. 4) through AMPK-dependent modulation of lipid metabolism. The reduced FAO in MCU KD astrocytes resulted in increased anaerobic glycolysis and extracellular lactate production thus, preserving neuronal function (Fig. 5).

Previous studies have shown cell death and dysfunction to be enhanced in other CNS cell systems by the synergistic effect of the HIV protein Tat and cocaine^1,26,60–63^. In line with other findings, our data also indicate that Tat and cocaine independently promote some aspects of a metabolic switch in astrocytes from neurotrophic to neurotoxic, but combined these factors function in an additive manner to induce a neurotoxic phenotype. In summary, upon activation of astrocytes by Tat/cocaine, the [Ca^2+^]_m_ uptake is augmented with a retrograde mROS generation and a positive feedback on MCU activity. The enhanced MCU activity modulates mitochondrial metabolism in astrocytes exposed to Tat/cocaine. Thus, by identifying a molecular link between astrocyte activation and mitochondrial metabolism this study provides a mechanistic connection for the etiology of HAND in the context of cocaine use and suggests promising therapeutic approaches.

## Electronic supplementary material

Supplemental Figure 1(PDF 498 kb)

Supplemental Figure 2(PDF 546 kb)

Supplemental Figure 3(PDF 3385 kb)

Supplemental Figure 4(PDF 5031 kb)

Supplementary Data 5(DOCX 181 kb)
