# Supplementary figures and images for: Astrocytic metabolic switch is a novel etiology for Cocaine and HIV-1 Tat-mediated neurotoxicity
Source: Cell Death Dis. 2018 Mar 16;9(4):415. doi: 10.1038/s41419-018-0422-3 (PMC5856787; doi:10.1038/s41419-018-0422-3)

**Figure S1**

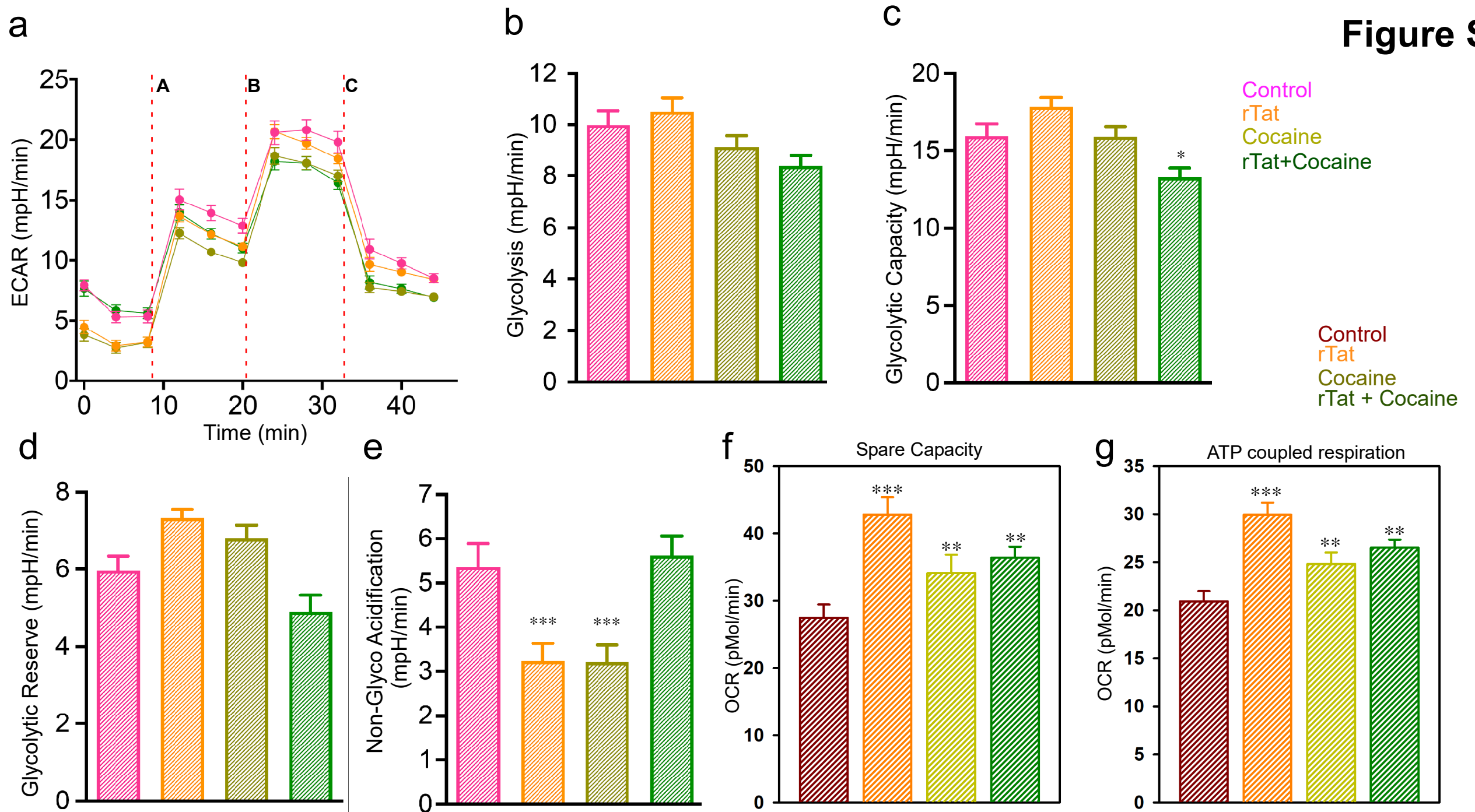

Supplement: Supplementary file 1 — Supplemental Figure 1(PDF 498 kb) [file 41419_2018_422_MOESM1_ESM.pdf]

# Figure S2

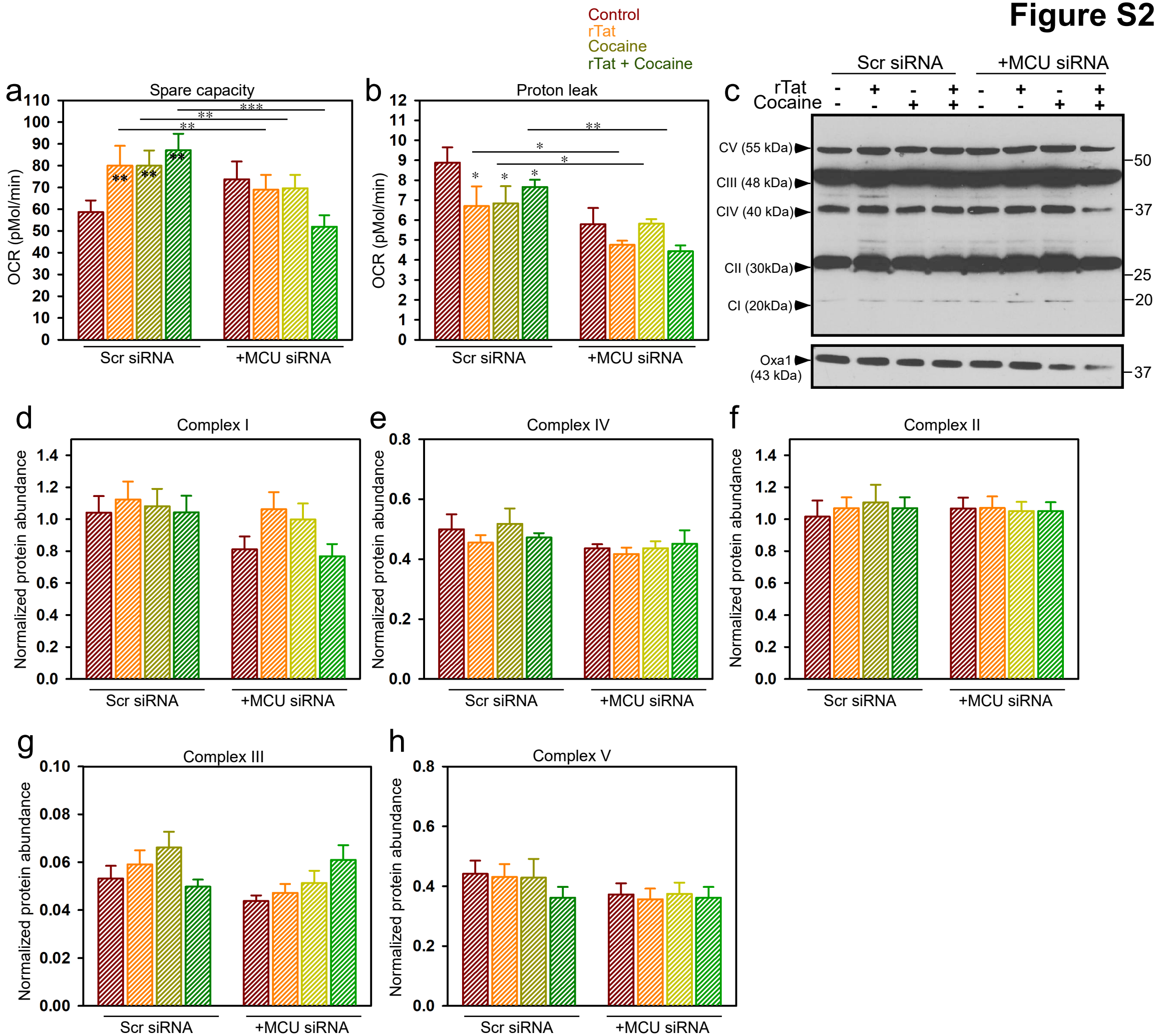

Supplement: Supplementary file 2 — Supplemental Figure 2(PDF 546 kb) [file 41419_2018_422_MOESM2_ESM.pdf]

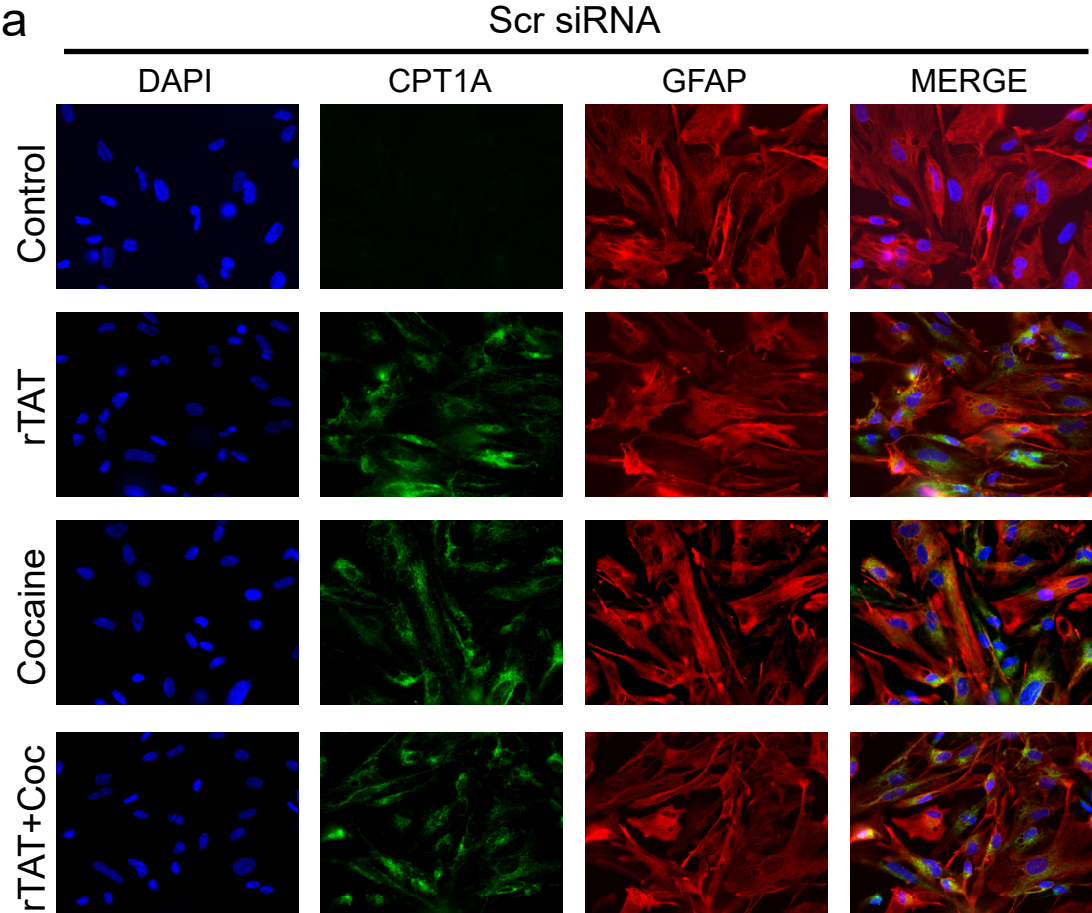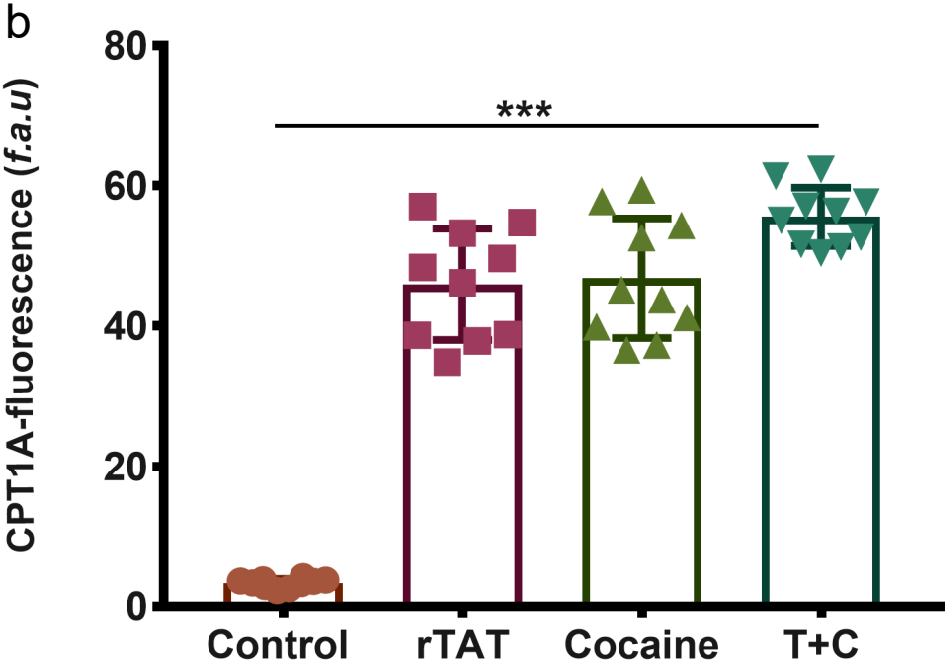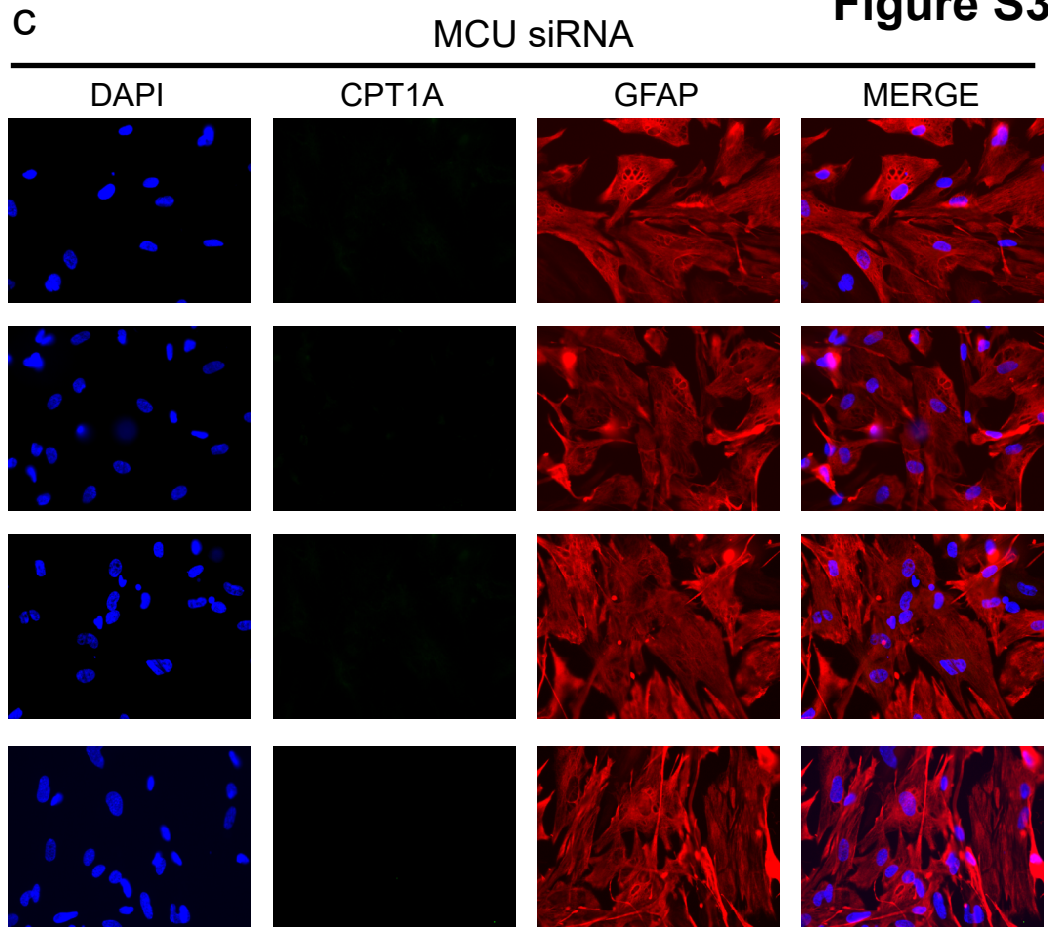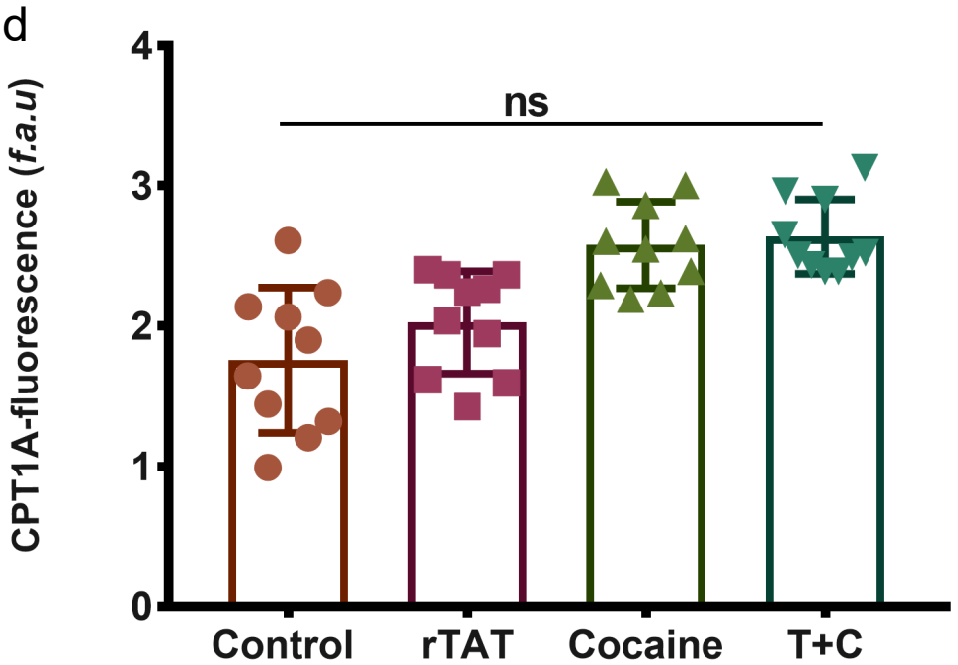

Supplement: Supplementary file 3 — Supplemental Figure 3(PDF 3385 kb) [file 41419_2018_422_MOESM3_ESM.pdf]

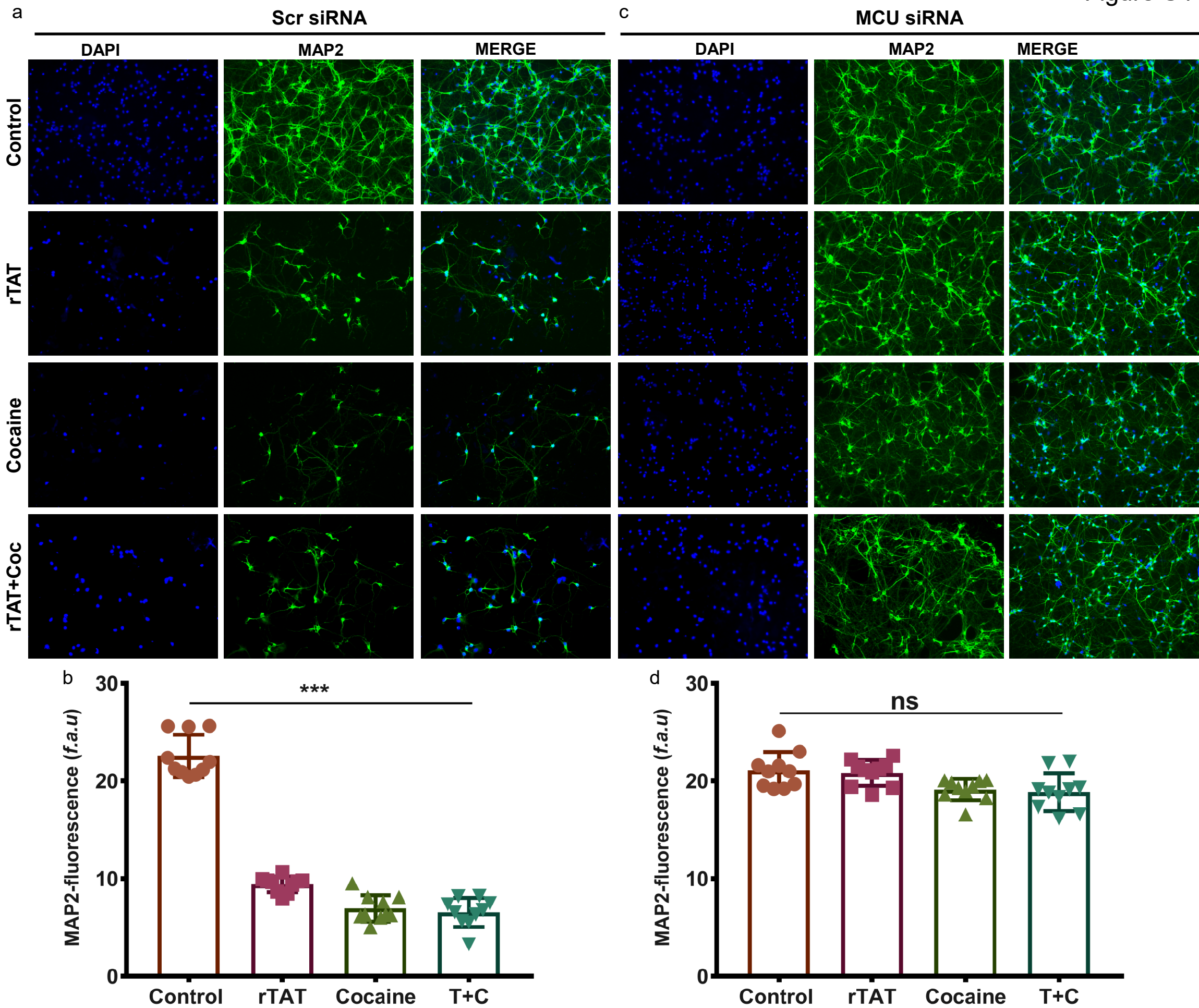

Supplement: Supplementary file 4 — Supplemental Figure 4(PDF 5031 kb) [file 41419_2018_422_MOESM4_ESM.pdf]
