# Supplementary material for: Astrocytic metabolic switch is a novel etiology for Cocaine and HIV-1 Tat-mediated neurotoxicity
Source: Cell Death Dis. 2018 Mar 16;9(4):415. doi: 10.1038/s41419-018-0422-3 (PMC5856787; doi:10.1038/s41419-018-0422-3)
Supplement: Supplementary file 5 — Supplementary Data 5(DOCX 181 kb) [file 41419_2018_422_MOESM5_ESM.docx]

**Astrocytic metabolic switch is a novel etiology for Cocaine and HIV-1 Tat-mediated neurotoxicity**

**Supplementary Information**

The supplemental data includes four Supplemental Figures with figure legends.

**SUPPLEMENTAL FIGURE LEGENDS**

**Figure S1: Glucose metabolism remains unaltered in astrocytes treated with rTat/Cocaine.** (**a**) Representative traces of ECAR measurements in astrocytes exposed to rTat, cocaine or rTat and cocaine. After basal measurement, glucose (A), Oligomycin (B), and 2DG (C) were added as indicated. (**b)** mean glycolysis, (**c**) glycolytic capacity, (**d)** glycolytic reserve, (**e)** and non-glycolytic acidification. (**f and g**) Quantification of spare capacity and ATP coupled respiration in astrocytes exposed to rTat, cocaine or rTat and cocaine. The spare capacity and ATP coupled respiration were calculated from Figure 1j. Data indicate Mean ± SEM; ***P <0.001, **P <0.01, * P <0.05; n=12-16.

**Figure S2. rTat/Cocaine augment MCU-mediated [Ca^2+^]_m_ Uptake.**

(**a**) Quantification of spare capacity and (**b**) proton leak in control (Scr siRNA) and MCU KD astrocytes exposed to rTat, cocaine or rTat and cocaine. The spare capacity and proton leak are calculated from Figure 3h and 3i. (**c**) Representative Western blot for electron transport chain (ETC) complex components in control (Scr siRNA) and MCU KD astrocytes exposed to rTat, cocaine or rTat and cocaine. Oxa1 was used as the loading control. The mitochondrial inner membrane protein Oxa1 was used as a loading control. (**d-h**) Quantification of the Complex I (**d**), Complex IV (**e**), Complex II (**f**), Complex III (**g**), and Complex V (**h**) protein levels in control and MCU KD astrocytes normalized to GAPDH. Data indicate Mean ± SEM; ***P <0.001, **P <0.01, * P <0.05; n=12-16.

**Figure S3. Knock down of MCU normalizes CPT1 levels and mitochondrial FAO.**

Control (**a**) and MCU-KD (**c**) astrocytes were treated with rTat/cocaine and the expression of GFAP and CPT1A levels were visualized by immunofluorescence. CPT1A fluorescence was quantified in control (**b**) or MCU KD (**d**) astrocytes treated with rTat/cocaine. 60X oil immersion images. Data represents Mean ± SEM; ***P <0.001; n = 6-8.

**Figure S4. Knock down of MCU in astrocytes protect neurons from rTat/Cocaine-induced toxicity.**

Neurons co-cultured with control (**a**) and MCU-KD (**c**) astrocytes were treated with rTat/cocaine and the microtubule network was visualized by immunofluorescence using anti-MAP-2 and GFAP antibody. MAP2 fluorescence was quantified in neurons co-cultured with control (**b**) or MCU KD (**d**) astrocytes treated with rTat/cocaine. 20X images. Data represents Mean ± SEM; ***P <0.001; n = 6-8.
